# A new pathogenic isolate of *Kocuria kristinae* identified for the first time in the marine fish *Larimichthys crocea*

**DOI:** 10.3389/fmicb.2023.1129568

**Published:** 2023-04-25

**Authors:** Xiangyu Meng, Fangyi Chen, Ming Xiong, Hua Hao, Ke-Jian Wang

**Affiliations:** ^1^State Key Laboratory of Marine Environmental Science, College of Ocean and Earth Sciences, Xiamen University, Xiamen, Fujian, China; ^2^State-Province Joint Engineering Laboratory of Marine Bioproducts and Technology, College of Ocean and Earth Sciences, Xiamen University, Xiamen, Fujian, China; ^3^Fujian Innovation Research Institute for Marine Biological Antimicrobial Peptide Industrial Technology, College of Ocean and Earth Sciences, Xiamen University, Xiamen, Fujian, China

**Keywords:** *Kocuria kristinae*, *Larimichthys crocea*, emerging pathogen, bacterial infection, whole genome sequencing

## Abstract

In recent years, new emerging pathogenic microorganisms have frequently appeared in animals, including marine fish, possibly due to climate change, anthropogenic activities, and even cross-species transmission of pathogenic microorganisms among animals or between animals and humans, which poses a serious issue for preventive medicine. In this study, a bacterium was clearly characterized among 64 isolates from the gills of diseased large yellow croaker *Larimichthys crocea* that were raised in marine aquaculture. This strain was identified as *K. kristinae* by biochemical tests with a VITEK 2.0 analysis system and *16S rRNA* sequencing and named *K. kristinae_LC*. The potential genes that might encode virulence-factors were widely screened through sequence analysis of the whole genome of *K. kristinae_LC*. Many genes involved in the two-component system and drug-resistance were also annotated. In addition, 104 unique genes in *K. kristinae_LC* were identified by pan genome analysis with the genomes of this strain from five different origins (woodpecker, medical resource, environment, and marine sponge reef) and the analysis results demonstrated that their predicted functions might be associated with adaptation to living conditions such as higher salinity, complex marine biomes, and low temperature. A significant difference in genomic organization was found among the *K. kristinae* strains that might be related to their hosts living in different environments. The animal regression test for this new bacterial isolate was carried out using *L. crocea*, and the results showed that this bacterium could cause the death of *L. crocea* and that the fish mortality was dose-dependent within 5 days post infection, indicating the pathogenicity of *K. kristinae_LC* to marine fish. Since *K. kristinae* has been reported as a pathogen for humans and bovines, in our study, we revealed a new isolate of *K. kristinae_LC* from marine fish for the first time, suggesting the potentiality of cross-species transmission among animals or from marine animals to humans, from which we would gain insight to help in future public prevention strategies for new emerging pathogens.

## 1. Introduction

Marine aquaculture is a necessary food resource and a main part of global trade, accounting for more than 50% of the total seafood supply (Okyere et al., 2018); fish can provide high-quality food containing proteins, vitamins, and saturated fatty acids. The large yellow croaker *L. crocea*, one of the most important economic species among the cultured fish in China, is characterized by its special physiological properties, food quality, and economic value. These fish are endemic marine fish that normally live in East Asia and belong to Sciaenidae in Perciformes. The major production area for *L. crocea* aquaculture and the main spawning center in China is located in coastal regions under the administration of Ningde city in Fujian Province. In recent years, the marine environment has become severely deteriorated due to human activities and complicated climate change, such as ocean warming and pollution, water eutrophication, and hypoxia, all of which affect the heathy development of aquaculture and threaten the diversity of fish (Lamb et al., 2018). Under the pressure of these factors, the microorganisms in the environment, including pathogenic microorganisms, might change in abundance or diversity in the community, or various pathogens may abruptly present in water, which often causes infectious diseases in humans or land animals (Carmona-Salido et al., 2021). Marine-farmed animals, including more than one hundred fish species, shrimp, crabs, etc., often suffer from various epidemic diseases that are usually caused by bacteria, viruses, or parasites, resulting in huge economic losses (Kühn and van Franeker, 2020). The pathogens that are generally considered common in aquaculture are *Vibrio* spp., *Pseudococcus* spp., and *Streptococcus* spp. in black seabreams and large yellow croakers, *Flavobacterium* spp. in rainbow trout, and *Edwardsiella* spp. in seabreams; furthermore, the fungal pathogens *Saprolegnia* spp. and the parasites *Trichodina*, *Chilodonela*, *Argulus*, and *Ergasilus*, as well as *Leeches* have been known for a long time (Kim et al., 2017; Buján et al., 2018; Duman et al., 2021; Abd El-Hack et al., 2022; Shahin et al., 2022; Ibrahim et al., 2022a,b). However, many unknown or emerging etiological diseases still appear frequently in aquatic farms, seriously affecting the development of aquaculture (Shahin et al., 2022). To date, a variety of bacteria that cause zoonotic diseases and emerging pathogenic bacteria have been reported in marine animals, including *Weissia ceti*, *Mycobacterium* spp., *Nocardia* spp., *Yersinia ruckeri*, *Francisella* spp., and *Lactococcus garvieae* (Vendrell et al., 2006; Gibello et al., 2016; Hashish et al., 2018; Chang et al., 2021; Del Rio-Rodriguez et al., 2021).

The genus *Kocuria* belongs to the family *Micrococcaceae*, suborder *Micrococcineae*, order *Actinomycetales*, class *Actinobacteria*. In the genus, eighteen species have been described so far: *K. varians*, *K. rosea*, *K. kristinae*, *K. palustris*, *K. rhizophila*, *K. marina*, *K. polaris*, *K. aegyptia*, *K. carniphila*, *K. himachalensis*, *K. flava*, *K. turfanensis*, *K. atrinae*, *K. gwangalliensis*, *K. halotolerans*, *K. koreensis*, *K. coralli*, and *K. salsicia* (Savini et al., 2010; Pulcrano et al., 2017; Li and Zhang, 2020; Pierron et al., 2021; Amadeo-Oreggioni et al., 2022). Most strains in the genus are pathogenic and anaerobic, and these bacteria have been reported to frequently appear on the skin, oral cavity, and urinary tracts of mammals, including humans, causing infections. Furthermore, these bacteria are always associated with immunocompromised patients and lead to more serious infections. Species like *K. rhizophila*, *K. kristinae*, *K. Endophthalmitis*, and *K. rosea* have been reported to cause infections in humans. *K. kristinae*, is also a common inhabitant of the skin and oral mucosa. It is a facultative anaerobic, nonmotile, catalase-positive, coagulase-negative, and Gram-positive coccoid action bacterium, which appears as tetrads or irregular clusters. Patients infected with this bacterium show different syndromes caused by other diseases. Although *K. kristinae* has always been considered a normal flora, it has recently been found to act as an etiological agent in a broad spectrum of human clinical diseases, such as peritonitis, endocarditis, cholecystitis, pneumonitis, and urinary catheter-related infectious diseases. In addition, it can also induce sepsis in certain cases where patients with immunodeficiency suffered from congenital tufting enteropathy, endocarditis, or central venous catheter-related bacteremia (Napolitani et al., 2019). It has been reported that patients with diabetes have a higher risk of being infected with *K. kristinae*, and that it can cause additional infections, such as retropharyngeal abscess and intracranial infection, stroke, suppurative thrombosis and septic pulmonary emboli, urinary tract infection, umbilical sepsis, and acute cholecystitis (Lin et al., 2019). Normally, this bacterium is isolated from blood, but it can also be found in other samples, such as peritoneal fluid, pus, sputum, synovial fluid, bile, fluid from abdominal abscesses, throat swabs, urine catheter tips, midstream urine, and pleural effusion (Kim et al., 2018; Živković Zarić et al., 2019). However, the bacterium *K. kristinae* has not yet been reported in marine animals. To date, there have only been two reports of the bacterium causing infections in bovines, and the strain was isolated from the vagina and reproductive tracts.

The purpose of the study was to identify the pathogenic bacteria from the cultured fish suffering from unknown diseases. Among those isolates of bacteria, one bacterium, *K. kristinae*, was cultured and identified, and it was reported for the first time to be isolated from *L. crocea*. Furthermore, the characteristics of this bacterium were elucidated in the study.

## 2. Materials and methods

### 2.1. Sample collection and pretreatment for bacterial cultivation

Samples in different tissues were separately collected from dying large yellow croakers with rotting lesions on the surface of their bodies, floating on the seawater (Supplementary Figure S1) in fish farms near Ningde city, Fujian Province, China, in 2018, but they had no obvious clinical manifestations in their organs after necropsy. Subsequently, organs including the heart, liver, spleen, head kidney, hind kidney, gills, fins, decayed skins, intestines, and brain were separately collected in 1 ml of 50% glycerinum diluted with 0.9% saline solution and then stored at −80°C.

### 2.2. Bacterial cultivation, isolation, and purification

For all the bacterial isolations, first, we washed the mentioned organs three times with sterilized heart and brain infusion (BHI) (Difco Becton Dickinson, United States) liquid medium and then homogenized them for further incubation at 37°C under aerobic and anaerobic conditions for 18 h at 180 rpm to harvest raw bacterial cultures. Second, we streaked the bacterial culture on BHI plates to obtain multiple colonies that might exhibit different colors, morphologies, sizes, or other visible features. Then, we picked each colony for serial purification by streaking it on the plates at least three times to ensure that all colonies showed identical features on each plate to obtain purified isolates. To further evaluate whether the new bacteria were hemolytic, they were cultured on Columbia blood agar plates (Hopebio, China).

### 2.3. Morphology observation by scanning electron microscopy and transmission electron microscopy

We employed SEM and TEM for further observation of the morphology, especially for *K. kristinae_LC*. The original bacterial culture of the strain was centrifuged (12,000 rpm for 2 min), and the supernatant was removed. The harvested cells were washed three times with 0.1 M phosphate buffer (pH 7.2), and then fixed in 2.5% glutaraldehyde solution (SINOPHARM, China) at 4°C overnight. Subsequently, the bacteria were dehydrated in a graded ethanol solution (SINOPHARM, China), dried at the critical point of CO_2_, mounted on metal stubs, coated with gold, and observed under a Zeiss Supra^™^ 55 scanning electron microscope for morphology observation. Bacterial cells were fixed overnight with 2.5% glutaraldehyde at 4°C. After washing with PBS three times, the samples were fixed with 1% osmic acid for 2 h. Then, the cells were washed with PBS three times, and dehydrated sequentially in ethanol at gradient concentrations (50, 70, and 90%) for 5 min at each gradient. Next, the cells were rinsed in 100% ethanol twice for 7 min. After dehydration, the cells were embedded in a resin at 25°C for 4 h. After polymerization at 65°C for 48 h, the samples were sliced and stained with uranyl acetate for 20 min, followed by alkaline lead citrate for 10 min. Finally, the prepared cells were observed under a transmission electron microscope (Hitachi HT-7800, Japan).

### 2.4. Amplicon sequencing of the *16S rRNA* gene and biochemical analysis

The full-length of *16S rRNA* gene sequence of this strain was 1,542 bp. To identify it, the universal PCR primers 27-F (5′AGAGTTTGATCCTGGCTCAG3′) and 1492-R (5′GGTTACCTTGTTACGACTT3′) were used for amplification of the *16S rRNA* gene. *16S rRNA* gene sequencing was performed following a standard protocol (An et al., 2006). Amplification was carried out on a thermal cycler with a preheating step of 98°C for 3 min, 30 cycles of denaturation at 98°C for 1 min, annealing at 58°C for 30 s, extension at 72°C for 1 min, and a final extension at 72°C for 5 min. The target PCR products were detected by 1% agarose gel electrophoresis and purified by a DNA purification kit (Tiangen, China). Sequencing of the PCR product was conducted at Sangon Biotech (Shanghai, PR China) Co., Ltd. Sequences were analyzed with the Basic Local Alignment Search Tool (BLAST, https://blast.ncbi.nlm.nih.gov/Blast.cgi), and an evolutionary tree was constructed by the neighbor-joining method in the MEGA 6.0 software package using 2,000 bootstrap replicates. The reference *16S rRNA* gene sequences were obtained from the genome by sequence alignment. For biochemical testing, the automated VITEK 2.0 Compact (C) (Biomeriux, North Carolina/United States) was employed for bacterial identification, using a Gram-positive GP REF 21342 identification (GPID) card according to the manufacturer’s instructions.

### 2.5. Genomic analysis of the *Kocuria kristinae_LC* strain based on whole genome sequence

#### 2.5.1. DNA extraction and genome sequencing

Genomic DNA of the strain was extracted using the hexadecyl trimethyl ammonium bromide (CTAB) (SINOPHARM, China) method according to the standard protocol. The qualified genomic DNA was fragmented with a G-tube (Covaris) and end-repaired to prepare a single molecule real time (SMRT) bell DNA template library (with a fragment size of >10 kb selected using the BluePippin system) according to the manufacturer’s specifications (PacBio, Menlo Park, CA). To evaluate the purity and concentration of genomic DNA and library reads, agarose gel electrophoresis was conducted to assess the integrity and purity of the genomic DNA (Supplementary Figure S2). Nanodrop and qubit were employed to measure the concentration of the genomic DNA (Supplementary Table S2) and the library reads (Supplementary Table S3). Furthermore, pulsed-field gel electrophoresis was carried out to assess the library reads quality (Supplementary Figure S3). SMRT sequencing was performed on a Pacific Biosciences RSII sequencer (PacBio, Menlo Park, CA) according to the standard protocols (MagBead Standard Seq v2 loading, 1 × 180 min movie) using the P4-C2 chemistry.

#### 2.5.2. *De novo* genome assembly

Continuous long reads were attained from three SMRT sequencing runs. Reads longer than 500 bp with a quality value of more than 0.75 were merged into a single dataset. Next, a hierarchical genome-assembly process (HGAP) pipeline was used to correct random errors in long seed reads (seed length threshold 6 kb) by aligning shorter reads from the same library (Chin et al., 2013). The obtained corrected pre-assembled reads were used for *de novo* assembly using the Celera Assembler website with overlapping layout consensus (OLC) strategy (Myers et al., 2000). Since SMRT sequencing features very little variations of the quality throughout the reads (Koren et al., 2012), no quality values were used during the assembly process. To verify the assembly quality and determine the final genome sequence, the Quiver consensus algorithm was used (Chin et al., 2013). Finally, the ends of the assembled sequence were trimmed to circularize the genome.

#### 2.5.3. Genomic prediction and annotations

The ORF was predicted using GeneMarkS (Besemer et al., 2001), which is a well-studied gene finding program for prokaryotic genome annotation. Several complementary approaches were applied to annotate the assembled sequences. The genes were annotated by aligning with the deposited ones in various protein databases including National Center for Biotechnology Information (NCBI) nonredundant protein (Nr), UniProt/Swiss-Prot, Kyoto Encyclopedia of Genes and Genomes (KEGG), Gene Ontology (GO), Cluster of Orthologous Groups of proteins (COG), and protein families (Pfam). Additional annotation was carried out based on the following databases: Pathogen Host Interactions (PHI), Virulence Factors of Pathogenic Bacteria (VFDB), Antibiotic Resistance Genes Database (ARDB), and Carbohydrate-Active enZYmes (CAZy). As described, prophage was predicted using PHAge Search Tool. Based on Nr annotation, GO annotation was carried out using Blast2GO and Pfam annotation was applied by Pfam_Scan.

### 2.6. Pathogenicity determination of the *Kocuria kristinae_LC* isolate by animal regression test

As no isolate of *K. kristinae* has been previously reported in marine fish previously, evaluation of whether this new bacterial isolate could cause infection or death of large yellow croakers was undertaken according to the method for animal regressive infection (Sibinga and Marquis, 2021; Yang et al., 2022). First, we streaked the reserved bacterial solution onto a BHI agar plate to obtain a single colony for further cultivation in BHI liquid medium at 37°C for 12 h at 180 rpm; then, we performed an extra inoculation from the obtained bacterial solution, which was at the logarithmic growth phase with an OD600.0 value of 1.874, at a ratio of 1:100 to prepare the bacterial culture. Next, the bacterial culture was centrifuged at 3,000 × g for 15 min to remove the supernatant and washed three times in a sterilized saline solution, and then the bacterial cells were resuspended in the washing solution. Finally, we intraperitoneally injected the fish with 2 × 10^8^ CFU, 2 × 10^7^ CFU, and 2 × 10^6^ CFU of this strain to check its pathogenicity. These infected fish were observed at 3 h, 6 h, 12 h, 24 h, 48 h, 72 h, 96 h, and 120 h post challenge. Fish survival curves and statistics were analyzed using GraphPad Prism 7 software (GraphPad Inc., San Diego, CA), and significance was determined by the Mantel-Cox log-rank test. *p* values below 0.05 were considered significant. In addition, paraffin sections of liver and spleen were stained with hematoxylin and eosin for histological analysis.

## 3. Results

### 3.1. Differential analysis of bacterial strains among all the isolates from diseased large yellow croakers

**Figure 1:**
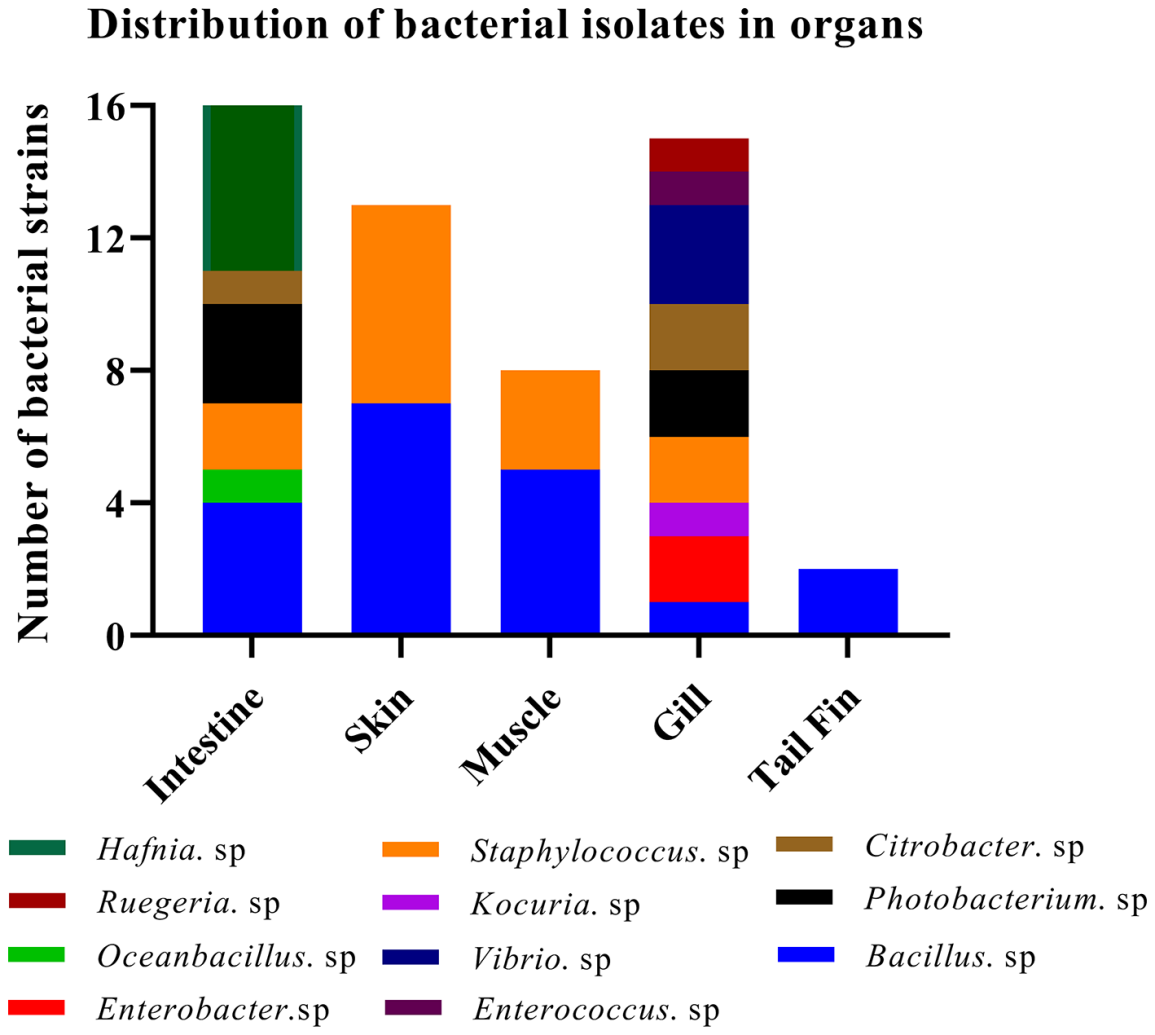
Identification of strains of 11 genera among 64 isolates from different organs of the diseased large yellow croaker.

A total of sixteen and twenty-four bacterial strains were isolated from organs in fish at 37°C under aerobic and anaerobic conditions, respectively. Similarly, twenty-four bacterial strains were obtained from organs at 27°C under anaerobic condition. Collectively, we successfully obtained fifteen *Hafnia* spp., nineteen *Bacillus* spp., two *Enterobacter* spp., one *Oceanbacillus*. sp., one *Kocuria*. sp., thirteen *Staphylococcus* spp., five *Photobacterium* spp., three *Citrobacter* spp., three *Vibrio* spp., one *Enterococcus*. Sp, and one *Ruegeria*. sp. Most strains have been reported to exhibit potential pathogenicity. Meanwhile, one bacterium, identified as *K. kristinae_LC*, was recognized for the first time in marine animals. The results indicated that a higher abundance and variety of bacterial species exist in the intestines and gills than in other organs. Summary data of the bacterial isolates are shown in Figure 1. By analyzing and collecting data from all the isolates from the organs, we found that the top three genera of bacteria are *Bacillus* spp., *Hafnia* spp., and *Staphylococcus* spp. with percentages of 29.69, 23.44, and 20.31%, respectively (Supplementary Table S5). Most bacteria have been reported to be zoonotic pathogens and to cause infection in humans, except *Oceanbacillus* sp. and *Ruegeria* sp.

### 3.2. Culture, isolation, and morphological identification of *Kocuria kristinae_LC*

**Figure 2:**
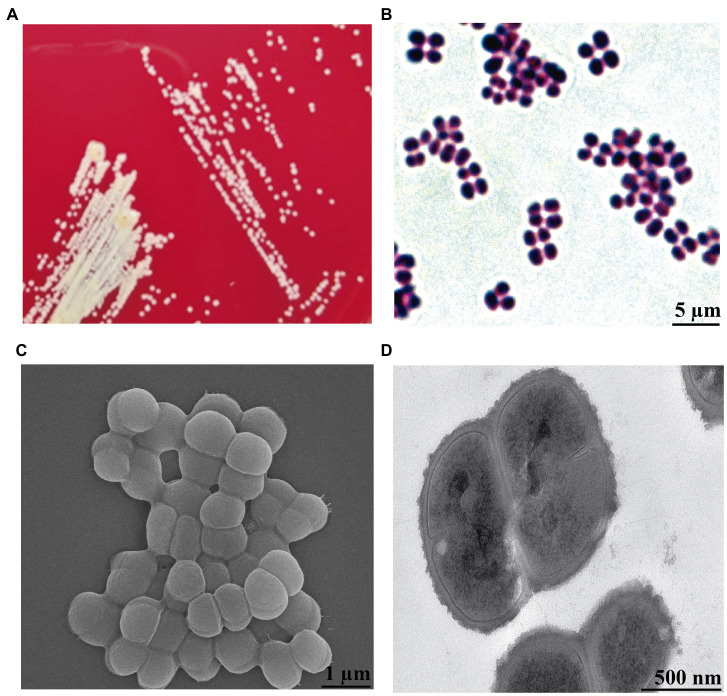
Morphology observation of *K. kristinae_LC* by cultivation, Gram staining, SEM and TEM. **(A)** Colonies of *K. kristinae_LC* on blood agar plates. **(B)** Gram staining of the bacteria was observed as blue-purple under microscopy, which demonstrated that this bacterium is Gram-positive. Morphology observation of the bacteria by SEM **(C)** and TEM **(D)**.

By analyzing the results of the isolated bacteria, we found a strain that was identified as *K. kristinae* through *16S rRNA* gene sequencing and biochemical tests. To our knowledge, *K. kristinae* has never been reported to be isolated from large yellow croakers. The strain was incubated overnight at 37°C, and colonies on blood agar plates showed small nonhemolytic colonies, which were creamish-white, opaque, round-convex with well-defined edges and matted texture. Gram staining of the colonies revealed the presence of Gram-positive cocci, most of which were arranged in tetrads (Figures 2A,B). To observe whether the bacteria exhibited some physical structures on the cell surface, a single colony was collected and cultured in BHI medium to prepare bacterial samples for further morphology observation by SEM and TEM. Under SEM, the spherical bacteria appeared smooth on the cell surface without fimbriae or flagellum, and tetrads of four to eight or even more cells were found (Figures 2C,D).

### 3.3. Phylogenetic evolutionary analysis and biochemical characterization of *Kocuria kristinae_LC*

**Figure 3:**
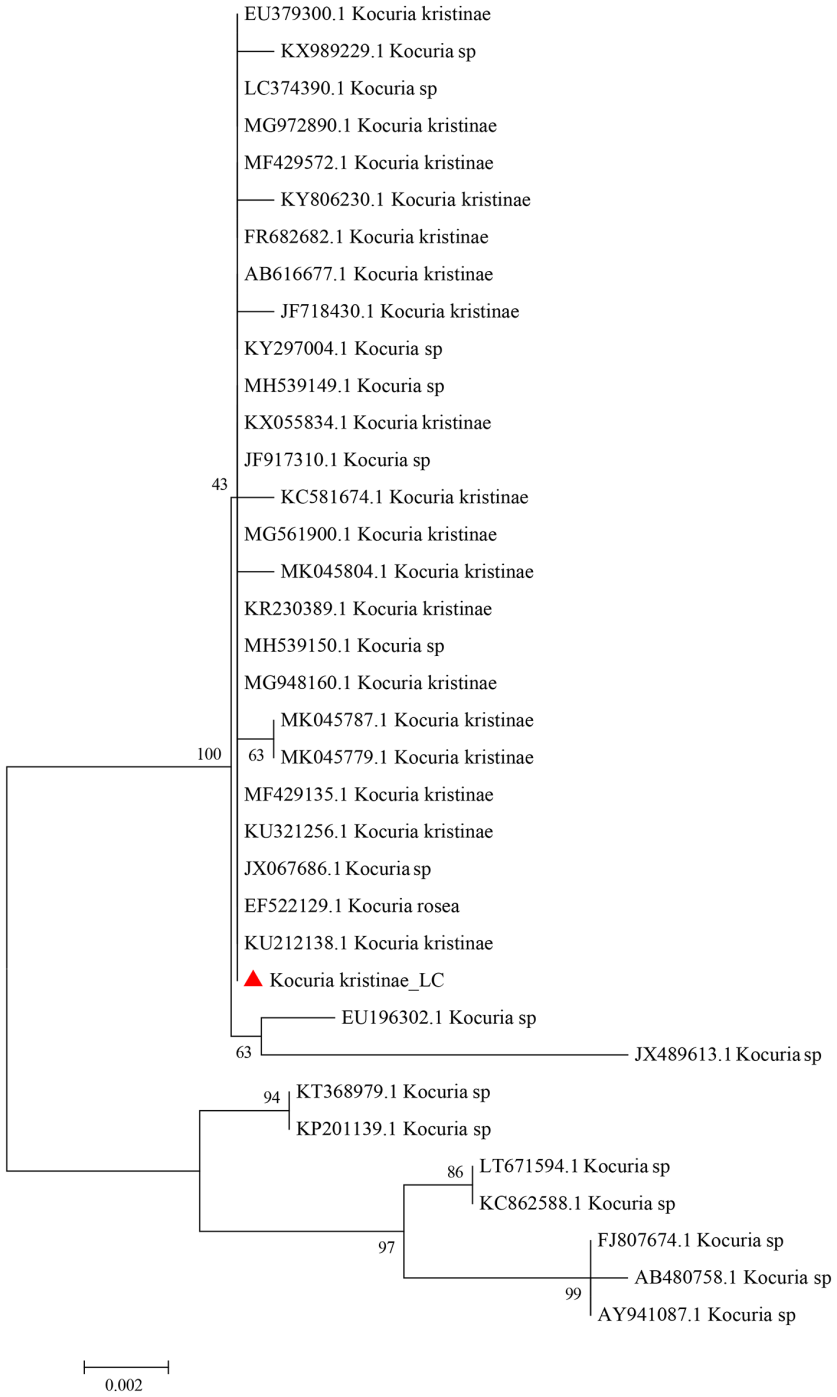
Phylogenetic evolutionary analysis of the new isolate *K. kristinae_LC* with other species. A phylogenetic tree was constructed based on the *16S rRNA* sequence by the N-J tree method in the MEGA 6.0 software package using 2,000 bootstrap replicas.

Based on the complete *16S rRNA* gene sequence, we conducted evolutionary analysis between this isolate and other *K. kristinae* strains. The results showed 99% similarity with those of multiple strains whose genes were submitted to the GenBank database. The evolutionary analysis showed that this strain was clustered in the same clade with most *K. kristinae* strains, which indicated that this isolate belonged to the *Kocuria* genus and shared the same evolutionary origin (Figure 3). This strain was identified as *K. kristinae* using a GPID card, revealing 99% species identification probability *via* the VITEK 2.0 system. The strain could produce type 1 arginine dihydrolase, alanine araminase, *α*-glucosidase, tyrosine araminase, leucine araminase, l-proline arylaminase, and pyrrole alkyl arylaminase. Additionally, it could utilize D–maltose, D–mannose, and sucrose. Meanwhile, this isolate could grow in a medium supplemented with 6.5% NaCl and showed resistance to optochin; all of the biological tests are shown in Table 1.

**Table 1:** Characterization of *K. kristinae_LC* by biochemical tests.

Biochemical tests							
02 AMY	−	04 PIPLC	−	05 dXYL	−	08 ADH1	+
09 BGAL	−	11 AGLU	+	13 APPA	−	14 CDEX	−
15 AspA	−	16 BGAR	−	17 AMAN	−	19 PHOS	−
20 LeuA	+	23 ProA	+	24 BGURr	−	25 AGAL	−
26 PyrA	+	27 BGUR	−	28 AlaA	+	29 TyrA	+
30 dSOR	−	31 URE	−	32 POLYB	−	37 dGAL	−
38 dRIB	−	39 ILATk	−	42 LAC	−	44 NA	−
45 dMAL	+	46 BACI	−	47 NOVO	−	50 NC6.5	+
52 dMAN	−	53 dMNE	+	54 MBdG	−	55 PUL	−
57 dRAF	−	58 O129R	−	59 SAL	−	60 SAC	+
62 dTRE	−	63 ADH2s	−	64 OPTO	+		

### 3.4. Genome assembly, annotation, and comparative analysis of the fish isolate *Kocuria kristinae_LC*

**Figure 4:**
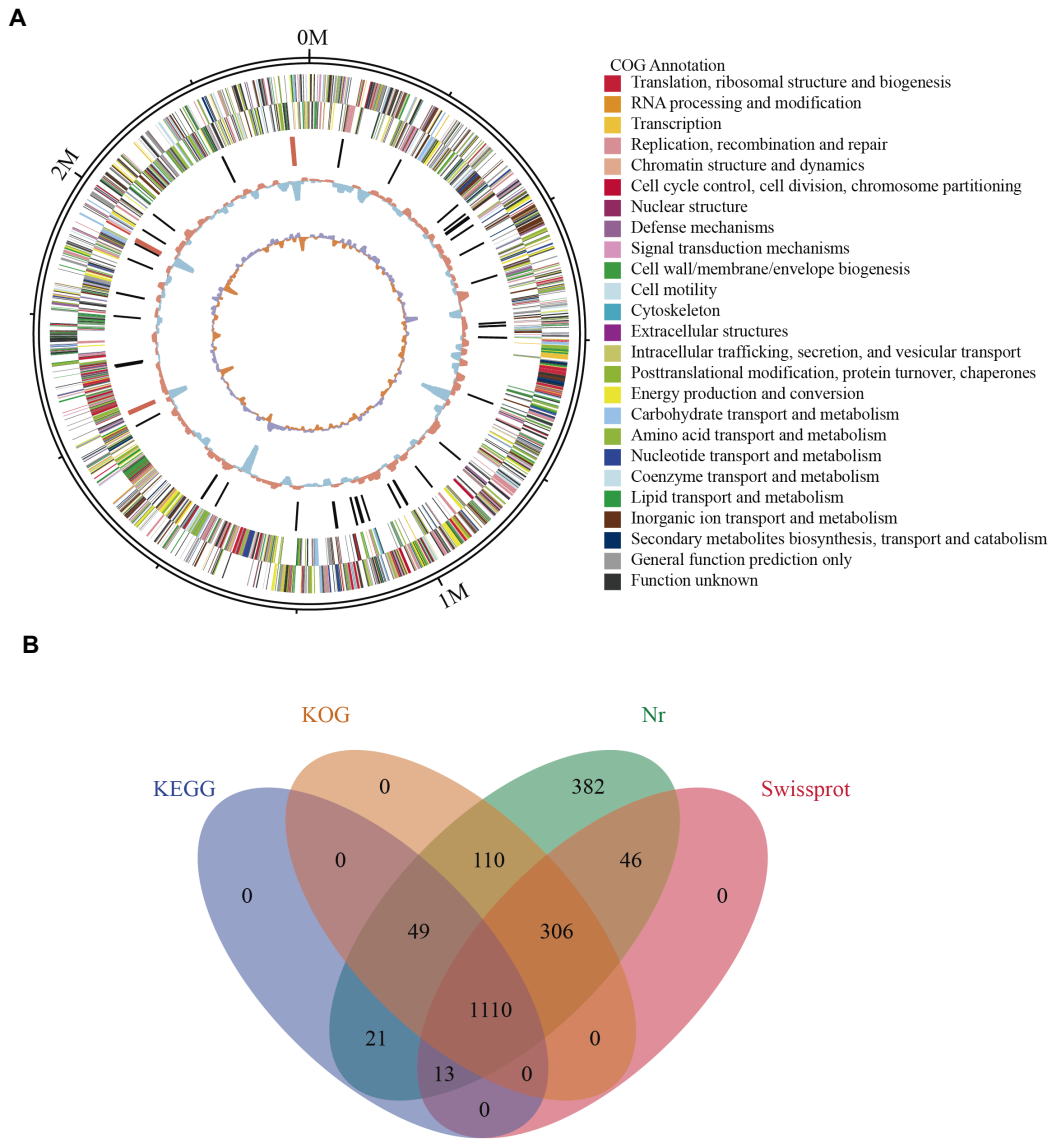
Genome assembly and annotation of *K. kristinae_LC*. **(A)** Circular representation of the genome of *K. kristinae_LC*. **(B)** Venn diagram showing shared and distinct orthologous genes for protein functions based on different databases.

Whole genome profiling analysis was conducted to better understand the pathogenesis of this strain and provide insights for further research. The results of the whole genome sequence of *K. kristinae_LC* revealed a genome sequence of 2,364,806 bp with an overall GC content of 71.75%, thereby indicating a circular chromosome structure. A total of 2,077 protein-coding genes, forty-seven tRNA genes, ten gene islands (GIs), three clustered regularly interspaced short palindromic repeats (CRISPRs), seventeen repeats, and fifty-seven noncoding RNAs were annotated. However, out of the genes encoding a total 2,077 proteins, sixty genes were predicted to be involved in the bacterial secretion system (Table 2). The circular genome map and summary characteristics of the strain are shown in Figure 4A and Table 2. Genes in the isolate genome that are related to drug resistance and virulence factors and genes that might play important roles in pathogenesis were analyzed. Generally, genes that might be involved in pathogenesis were predicted, including 298 genes encoding ATP binding cassette (ABC) transporter permeases, twenty genes encoding hemolysin, twenty-two genes encoding multiple drug transporter ATPases, forty-six genes encoding multidrug ABC transporter ATP-binding proteins, and eight genes directly annotated to encode virulence factors. Based on the whole genome, different annotation results were suggested by reviews of different databases (Figure 4B). Through the COG database, twenty-nine genes were found to be associated with defense mechanisms, and eight genes were putative virulence factors belonging to the outer membrane protein family. Thirty-seven genes encoding virulence factors that are predicted to be involved in mouse systematic infections were identified through the PHI database. Three genes essential to virulence and twenty-one related genes involved in the secretion system were also detected. According to the VFDB database, twenty-one virulence factors were detected and showed high homology with *M. abscessus*. Furthermore, we also predicted sixteen genes with characterized functions that showed closer similarity with *M. abscessus*. Meanwhile, according to the antibiotic resistance gene database, seven antibiotic resistance genes were predicted, and this isolate was predicted to be resistant to bacitracin.

**Table 2:** Whole genome assembly features of the *K. kristinae_LC*.

Whole genome assembly	*K. kristinae_LC*
Total genome size(bp)	2,364,806
GC content (%)	71.75
Number of tRNA	47
Number of CDS	2,077
Number of GIs	10
Number of CRISPR	3
Number of repeats	17
Total number of annotated proteins	2,077
Total number of secretion proteins	60
Total number of non-secretion proteins	2,017

**Figure 5:**
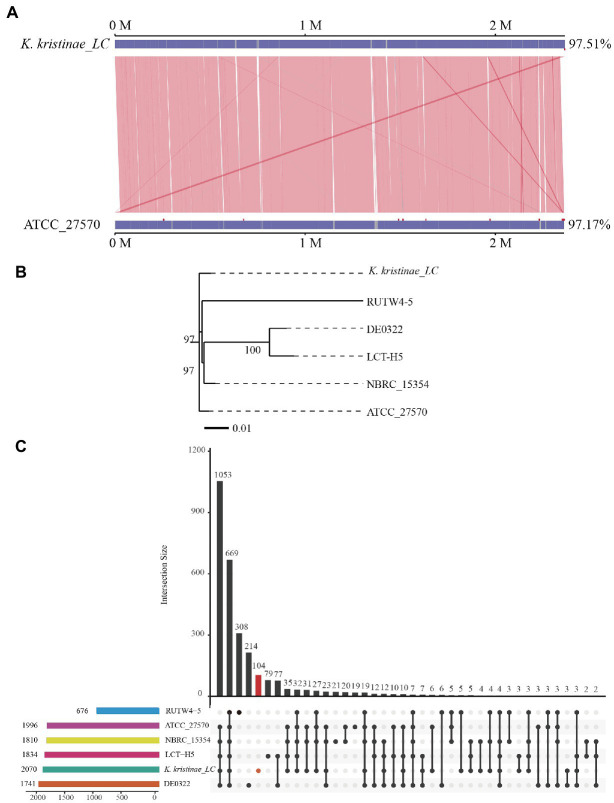
Comparative analysis of *K. kristinae_LC* with other strains. **(A)** Linearity analysis of *K. kristinae_LC* and ATCC_27570. **(B)** A phylogenetic evolutionary tree was constructed for *K. kristinae_LC* and other strains from different animals or origins. **(C)** Pangenome analysis of *K. kristinae_LC* with other isolates from different hosts for homology gene and distinct gene detection.

To reveal the initial origin and possible transmission route between fish and other animals of the strain, comparative genome analysis is necessary and was conducted. Collinearity analysis was conducted based on the genome sequences of this strain and *K. kristinae* ATCC 27570. The results showed that the proportion of collinear genes between these two strains accounted for 97.51% of the *K. kristinae_LC* genome and 97.17% of the *K. kristinae* ATCC 27570 genome (Figure 5A). Based on the whole genome sequence, a phylogenetic evolutionary tree was constructed and indicated that *K. kristinae_LC* showed higher homology and significant base differences with other *K. kristinae* strains, revealing high conservation and plasticity (Figure 5B). To further understand the potential pathogenesis between the strains, pangenome analysis was conducted to detect whether certain specific genes exist in this isolate that might contribute to infection progression. Notably, 104 specific genes were detected in the genome of *K. kristinae_LC* whose functions are associated with cellular processes, including ion transporters, enzymes or amino acid metabolism, cell division, transcriptional regulators, and housekeeping genes, such as genes encoding RNA polymerase sigma subunits. Considering the complex environment in which the bacterial isolate lives, such as higher salinity, complex marine biomes, and lower temperature in the ocean, it is speculated that these genes might be coordinated to maintain the normal physiological processes for the bacterium, which may be different from other strains. Among the 104 genes, four were also found in the PHI database, namely, gene_ 0336, gene_ 0781, gene_1280, and gene_1957, encoding cystathionine beta-lyase, polyketide synthetase proteins, multidrug resistance proteins, and posttranscriptional regulators, respectively, (Figure 5C).

### 3.5. The new isolate of *Kocuria kristinae_LC* showed potent pathogenicity to the marine fish *Larimichthys crocea*

**Figure 6:**
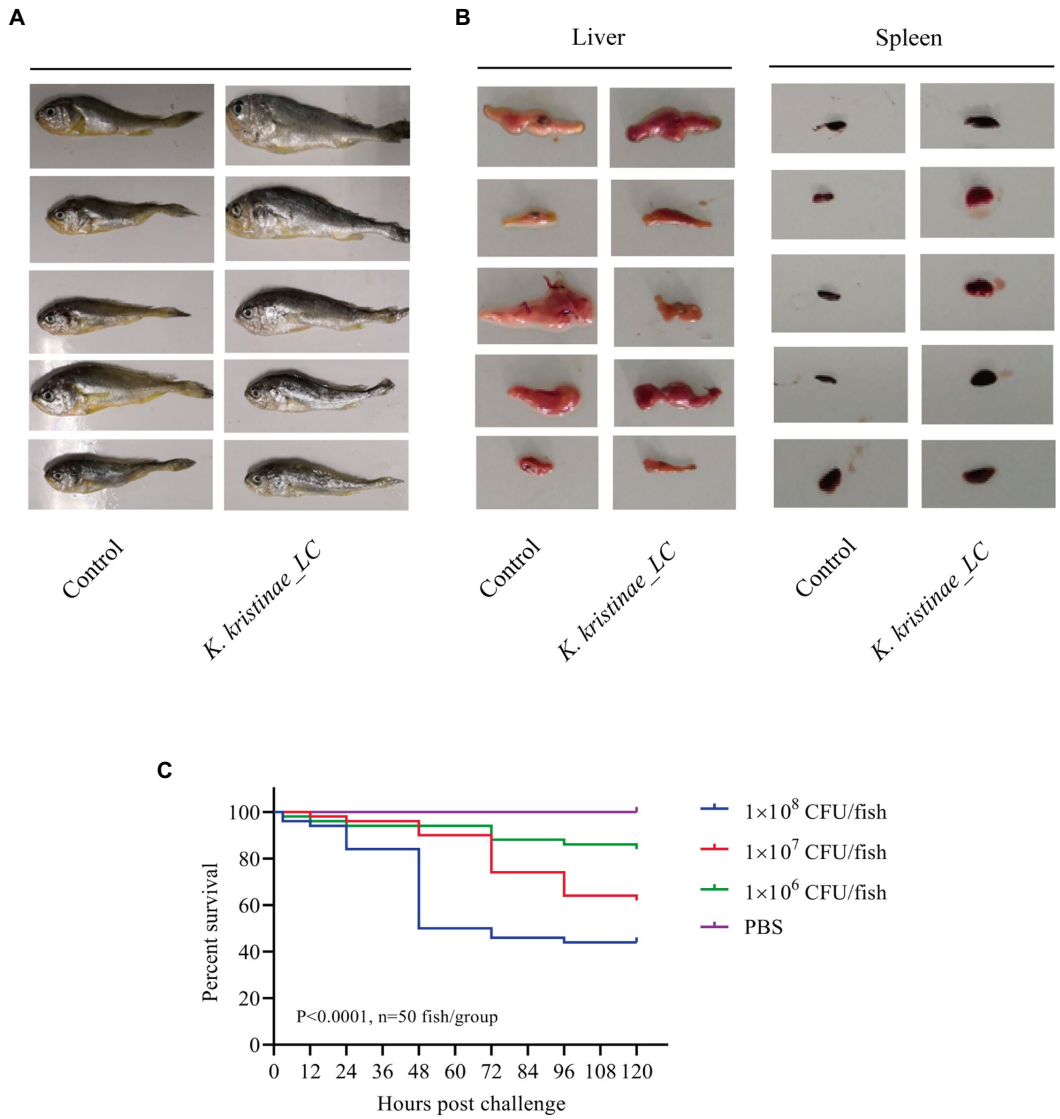
Pathological observation in large yellow croaker infected with *K. kristinae_LC*. The infected fish did not show manifestations on the body **(A)** but showed hemorrhage and swelling in the liver and spleen. Photographs of large yellow croaker organs in the regressive infection experiment **(B)**. Fish were infected intraperitoneally with 2 × 10^8^ CFU, 2 × 10^7^ CFU, or 2 × 10^6^ CFU of *K. kristinae_LC*. The survival percentage was recorded for 120 h **(C)**, and significance was assessed by the log-rank Mantel–Cox test (*p* value indicated on the graph; *n* = 50 fish per group).

**Figure 7:**
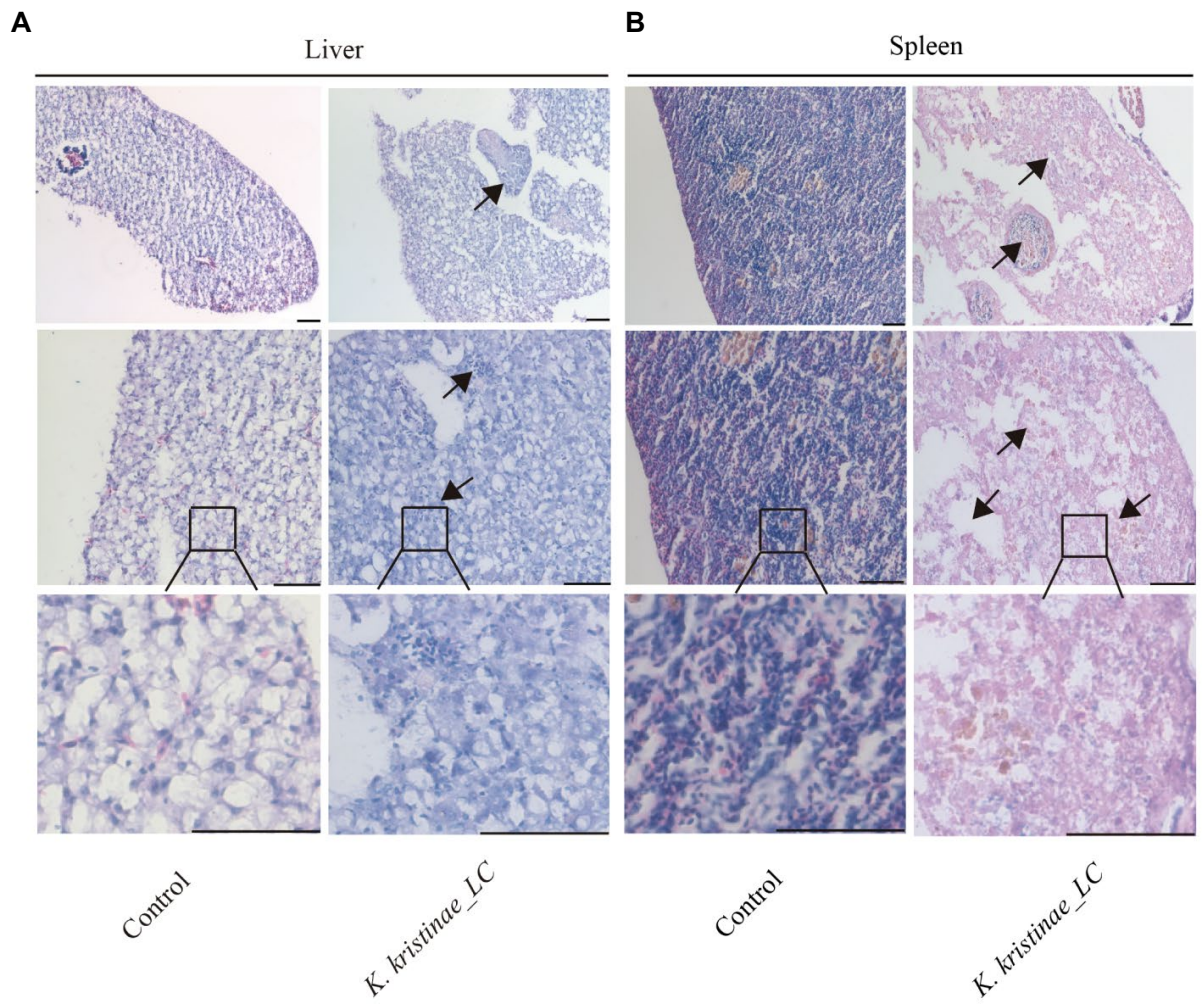
Histopathological observation of the liver and spleen infected with *K. kristinae_LC*. H&E-stained slices of the infected fish organs at 48 h post-infection for liver **(A)** and spleen **(B)**. The scale bar is 50 μm.

The results showed that these fish died after challenge with these bacteria at different CFUs, even without any pathological manifestations on the body surface (Figure 6A). These infected fish were monitored for 5 days, and the mortality of each administered group was dose-dependent. The *in vivo* results showed that for intraperitoneal inoculation of three doses of bacteria, 2 × 10^8^ CFU, 2 × 10^7^ CFU, and 2 × 10^6^ CFU, the mortality rate at 120 h post challenge was 56, 38, and 16%, respectively. During the experiment, these infected fish were observed at 3 h, 6 h, 12 h, 24 h, 48 h, 72 h, 96 h, and 120 h post challenge, and they did not die until the 12-h time point. The main period of infection for this bacterium was from 36 h to 48 h post infection (hpi). The time point with the highest mortality rate was 48 hpi, after which the mortality rate decreased until no death occurred at 120 h. Later, these fish were dissected, and the experimental group showed obvious symptomatic manifestations in which the liver and spleen exhibited hemorrhage and swelling (Figure 6B), and we recovered the bacteria from the liver and spleen (Supplementary Figure S4). The survival rate is shown in detail for the different time points for the experimental and control groups in the survival plot (Figure 6C). From histopathological tests, we observed disintegration of the hepatocyte cord in the liver, infiltration of lymphocytes, and necrosis in hepatocytes (Figure 7A). Additionally, we found that the splenic structure disappeared, the medullary cord ruptured, and lymphocytes were necrotic, thereby exuding intracellular fibrins (Figure 7B).

## 4. Discussion

The epidemiological characteristics of zoonosis always show cross infection between animals and humans, thereby posing a serious threat to public health. In recent years, it has been reported that some common and emerging bacterial strains isolated from diseased marine fish have homologies with pathogens that were originally found in human infections (Rodriguez et al., 2016), such as *V. parahaemolyticus* (Guin et al., 2019), *S. agalactiae* (Kalimuddin et al., 2017), *H. pylori* (Abdel-Moein et al., 2015), *Aeromonas* spp. (Borella et al., 2020; Ibrahim et al., 2022a,b), *E. coli*, *Shigella* spp., *Salmonella* spp., *L. monocytogenes*, *Weissella* spp. (Castrejón-Nájera et al., 2018), *Rahnella* spp. (Lamb et al., 2018), *Yersinia* spp. (Guijarro et al., 2018; Wrobel et al., 2019), and *Francisella* spp. (Birkbeck et al., 2011). Although most of the strains are opportunistic pathogens in non-fish species, once fish are infected with these bacteria, they can certainly have a negative impact on the fish and even lead to fish disease or death, which can result in economic losses. Over the years, researchers have developed measurements, vaccines, and feed additives (clove oil, quercetin, cinnamaldehyde, microalgae, thymol, and thymoquinone) for direct bacterial disease prevention and have even developed positive regulation strategies associated with the immune system in fish, which have contributed to healthy fishery development, but great economic losses still occur due to unknown reasons. (Abd El-Hamid et al., 2016; Chatakondi et al., 2018; Abd El-Hamid et al., 2021; Bøgwald and Dalmo, 2021; Ibrahim et al., 2021; Santos et al., 2021). It is speculated that some unknown pathogens have not been diagnosed or that emerging bacterial pathogens may show pathogenicity to marine animals.

In this study, we focused on isolating new bacterial pathogens from diseased fish that may be potential pathogens causing some infectious diseases in marine animals. During the bacterial isolation experiment, we obtained sixty-four purified isolates under different conditions. By analyzing the collective data of all the isolates from the organs, we found that the top three bacterial genera were *Bacillus* spp., *Hafnia* spp., and *Staphylococcus* spp. with percentages of 29.69, 23.44, and 20.31%, respectively. Most of the bacteria have been reported to be zoonotic pathogens and to cause infections in humans, with the exception of *Oceanbacillus* sp. and *Ruegeria* sp., suggesting a potential fish-to-human bacterial transmission (Savini et al., 2009; Bandeira Junior et al., 2018; Terceti et al., 2019; Yin et al., 2019; Cao et al., 2021; Enosi Tuipulotu et al., 2021; Ribeiro et al., 2021; Al-Eqabi et al., 2022; Alzahrani et al., 2022; Choi and Choi, 2022; Ionescu et al., 2022). Among these known pathogens in marine fish, one unreported bacterial strain in the marine-cultured fish *L. crocea* was characterized and named *K. kristinae_LC*. In most reports, *K. kristinae* has been considered to be a common inhabitant of the skin or oral mucosa, and it has been isolated from cornea, pleural effusion, and peripheral blood samples in humans (Kim et al., 2018; Kate et al., 2020) and in two reports, the bacterium was isolated from the vaginal and reproductive tracts in bovines (Styková et al., 2013, 2016). Interestingly, in our study, isolation and culture of this bacterium from marine fish gills was demonstrated for the first time. We did not consider this bacterium to occur only in the skin or oral mucosa of fish, but it does commonly exist in mucosa. This is the first report of the isolation of this bacterial strain from aquatic animals. Therefore, we do not consider it to be particularly or universally present in any species of fish but tend to regard it as an emerging or transboundary causative agent of disease in marine fish. Furthermore, we isolated the strain from the gills of marine fish, which indicated that the bacterium might be present in different mucosal sites of different animals, not only in the oral mucosa and vaginal and reproductive tracts. The bacterium was previously described as a catalase-positive, coagulase-negative, nonmotile, Gram-positive facultative anaerobe that occurs in tetrads, which is consistent with our findings (Bernshteyn et al., 2020). To better identify and explore which virulence factors may contribute to its pathogenesis, whole genome profiling was conducted. By analyzing the genome annotation results, genes involved in the *AI-2E* transporter were detected. Based on the published literature, the *AI-2E* transporter plays a vital role in Na^+^(Li^+^)/H^+^ antiport activity and the pH response, which provides possibilities for bacteria to adapt to high saline-alkaline living conditions and participate in biological activities *via* quorum sensing-mediated communication, such as biofilm formation, enzyme secretion, virulence production, and signaling molecules (Wang et al., 2020). Some genes encoding the relaxase, a metal-dependent nuclease, that can break and integrate DNA fragments into the conjugative bacterial genome, causing horizontal gene transmission, especially for antibiotic resistance-associated genes, were also predicted (Pluta et al., 2017). In the PHI database, approximately 100 genes were predicted to be involved in resistance to multiple antibiotics. Meanwhile, two *relA* genes were also detected to encode virulence regulators in the genome of this isolate through VFDB. Based on the previous documents, we learned that deletion of the *relA* gene could abrogate the capability of bacteria to cause persistent infection, demonstrating an important role of these genes in destroying the host immune response (Abdellrazeq et al., 2020). Two genes encoding abortion infection proteins (also present in *Arthrobacter* spp.) were detected, and twenty-eight genes encoding uncharacterized proteins were predicted to be associated with the formation of abscesses and showed high similarity with *M abscessus*. In the genome of *K. kristinae_LC*, one questionable and two credible CRISPRs were predicted, which are involved in the natural immune system in bacteria (Moon et al., 2019). Apart from these important CRISPRs, four genes encoding highly conserved Cas proteins, Cas4/Cas1 and Cas2, were also screened, which are common to almost all CRISPR-Cas systems, and these conserved adaptive regulatory proteins could form an integrase complex. The CRISPR-Cas system provides a foundation to ensure the integral nature of different genes in the bacterial genome for normal functions, such as functions as enzymes, regulators, and virulence factors (Makarova et al., 2017; Xiao et al., 2017). Genome-wide profiling analysis revealed four genes encoding prevent-host-death proteins, which showed important significance during cell development. Once these genes are overexpressed, they can contribute to antibiotic resistance and increase the level of biofilm formation (Petrova et al., 2011). Notably, these genes have also been found in *K. kristinae_LC*. The pathogenesis of this strain is still unknown, and we still need to conduct more research to validate the functions of these predicted genes, especially those that might contribute to infection progression. From the comparative genome analysis, it was confirmed that this strain also shared many genes with other strains in the genome that were isolated from the environment or medical materials; it was even isolated from woodpeckers. Thus, it was suspected that a coinfection or transboundary transmission might occur from land animals or birds carrying pathogenic microorganisms to the aquatic animals (Ayyal Al-Gburi, 2020). Pangenome analysis results also revealed 104 genes whose functions are associated with adaptation to living conditions with higher salinity, complex marine biomes, or lower temperature, thus improving bacterial growth. As reported, most strains in the *Kocuria* genus have been found to cause different infections in humans, such as *K. marina* (Pulcrano et al., 2017), *K. rhizophila*, *K. kristinae*, *K. Endophthalmitis* (Amadeo-Oreggioni et al., 2022), *K. varians* (Grama et al., 2021), and *K. rosea*. As for *K. kristinae*, only two reports have demonstrated that it could be isolated from bovine reproductive and vaginal tracts. And *K. marina* was reported as an emerging pathogen in wild rats (Loong et al., 2016). In aquatic animals, we only found that *Kocuria* genus strains were isolated from sponge (Palomo et al., 2013) and coral mucus (Palermo et al., 2016), and in these studies, they did not accurately identify the strains or indicate that the isolates could cause infection or death. And only one literature showed that *K. sediminis* was isolated from marine sedimental samples. So, we suspected that *K. kristinae* might originate from terrestrial animals and cause cross-species infections or act as an opportunistic pathogen in the environment.

Considering that this isolate was obtained from gills, which are completely exposed to the marine environment, we speculated that the strain possibly originated from a land environment, which corresponds to the phylogeny tree analysis based on the *16S rRNA* gene sequence. High homology and genome plasticity was observed between *K. kristinae_LC* and other strains, namely, RUTW4-5 (marine sponge reef), ATCC 27570 (woodpecker), NBRC_15354 (medical resource), LCT-H5(environment), and DE 0322 (environment), indicating the possibility of transboundary transmission and explaining the genomic differences between strains, which is due to bacterial living conditions. Based on the comparative genome analysis, it was speculated that some medical materials or wasted water produced in hospitals were discharged without being thoroughly sterilized, causing pathogen spreading and negative impacts on the environment. Traditional pathogens in fish diseases are mainly classified as Gram-negative bacteria, and the isolation of this Gram-positive germ provides new insights for the prevention and control of fish diseases. Verification of whether diseases caused by this bacterium can be spread through polluted water, medical wastes, feces, and contaminated food requires much work. Furthermore, we should also note that infected fish showed no symptoms on the body surface in our research, which is unexpected, and once these fish act as carriers of this bacteria, it will put humans or other animals that feed on this kind of fish in danger (Ayyal Al-Gburi, 2020). We need to conduct a massive amount of future research to determine, for instance, whether new pathogens have the potential to cause transboundary transmission between different hosts. Most importantly, we should spend more effort and time establishing genomic manipulation methods for these new pathogens to verify the functions of genes that might participate in the pathogenesis of etiological bacteria.

Because this is the first study to identify the isolates in *L. crocea*, the animal regression test was used to further confirm that the new bacterial isolates were important, and the results, as expected, showed that the new isolate *K. kristinae_LC* can cause death in *L. crocea*. When these fish were inoculated with different doses of bacteria intraperitoneally, dose-dependent mortality was observed. The regressive infection results demonstrated that this new isolate showed pathogenicity to the tested large yellow croaker. In the Figure 1, we collected the data of bacteria isolated from diseased fish and found that most isolated strains could cause infection in marine animals or aquatic animals, including large yellow croakers. As reported, the bacteria could cause infection or death in fish without obvious manifestations, except for hemorrhage in organs, and our results were similar to those of previous reports (as shown in Figure 6). Regarding the original samples, we collected diseased fish in October, 2018, when the seawater temperature was approximately 28°C, at which point diverse pathogens thrive. The fish might become infected with different pathogenic bacteria that cause different clinical symptoms of infections; that is, fish which are farmed in cages in the ocean at high breeding densities often suffer from body lesions or skin decay, which might provide access to some other pathogenic microorganisms. In fact, we did not obtain any isolate of *K. kristinae* from the samples of rotten skin or damaged tissue in fish but only identified this bacterium in gills; thus, no lesions observed in experimental infections with *K. kristinae* in monocultures were different from those observed in polymicrobial infections. It is important to note that when *K. kristinae* caused infection, it always occurred in opportunistic situations in patients who shared catheter-related diseases or immune-compromised characteristics.

In summary, we successfully cultured sixty-four isolates from diseased *L. crocea* organs, among which a bacterium was clearly identified as *K. kristinae* by biochemical tests and *16S rRNA* sequencing and was named *K. kristinae_LC*. From the morphology observation, the bacterium was Gram-positive and appeared as tetrads or irregular clusters. To the best of our knowledge, this is the first report of the bacterium being isolated from marine fish, and whole genome sequencing was conducted to better understand the features of the isolate. The potential genes associated with virulence factors were widely screened through sequence analysis based on the whole genome. Furthermore, unique genes in *K. kristinae_LC* were identified by pangenome analysis with genomes from other strains of different origins, and the analysis results demonstrated that their predicted functions might be associated with adaptation to living conditions, such as higher salinity, complex marine biomes, and low temperature. A significant difference in genomic organization was found among the *K. kristinae* strains that might be related to their hosts living in different environments. Importantly, the animal regression test for the new bacterial isolate showed that this bacterium could cause death of *L. crocea* and the fish mortality was dose-dependent within 5 days post infection, indicating the pathogenicity of *K. kristinae_LC* to marine fish. All these data provide insight for future public prevention of new emerging pathogens.

## Data availability statement

The datasets presented in this study can be found in online repositories. The names of the repository/repositories and accession number(s) can be found in the article/Supplementary material.

## Ethics statement

The animal study was reviewed and approved by the Laboratory Animal Management and Ethics Committee of Xiamen University.

## Author contributions

K-JW: conceptualization, funding acquisition, project administration, supervision, and writing-review and editing. FC: funding acquisition, project administration, supervision, and writing-review and editing. XM: sample collection and performing the experiments, data curation, formal analysis, and writing-original manuscript. MX and HH: sample collection. All authors contributed to the article and approved the submitted version.

## Funding

This study was supported by the National Natural Science Foundation of China (grant #U1805233), the Natural Science Foundation of Fujian Province, China (grant #2021J05008), Marine Biotechnology Economic Integration Service Platform from Fujian Association for Science and Technology, the Xiamen Ocean and Fishery Development Special Fund Project (grant #20CZP011HJ06) from the Xiamen Municipal Bureau of Ocean Development, and a grant (grant #3502Z20203012) from the Xiamen Science and Technology Planning Project.

## Conflict of interest

The authors declare that the research was conducted in the absence of any commercial or financial relationships that could be construed as a potential conflict of interest.

## Publisher’s note

All claims expressed in this article are solely those of the authors and do not necessarily represent those of their affiliated organizations, or those of the publisher, the editors and the reviewers. Any product that may be evaluated in this article, or claim that may be made by its manufacturer, is not guaranteed or endorsed by the publisher.
