# Supplementary material for: A new pathogenic isolate of Kocuria kristinae identified for the first time in the marine fish Larimichthys crocea
Source: Front Microbiol. 2023 Apr 25;14:1129568. doi: 10.3389/fmicb.2023.1129568 (PMC10167289; doi:10.3389/fmicb.2023.1129568)
Supplement: Supplementary file 1 [file Data_Sheet_1.pdf]

A new pathogenic isolate of *Kocuria kristinae* identified for the first time in the marine fish *Larimichthys crocea*

Xiangyu Meng [1], Fangyi Chen [1,2,3], Ming Xiong[1,2,3], Hua Hao[1,2,3] and Ke-Jian Wang [1,2,3*]

[1]State Key Laboratory of Marine Environmental Science, College of Ocean and Earth Sciences, Xiamen University, Xiamen, China
[2]State-Province Joint Engineering Laboratory of Marine Bioproducts and Technology, College of Ocean and Earth Sciences, College of Ocean and Earth Sciences, Xiamen University, Xiamen, China
[3]Fujian Innovation Research Institute for Marine Biological Antimicrobial Peptide Industrial Technology, College of Ocean and Earth Sciences, Xiamen University, Xiamen, China

***Correspondence:**

Ke-Jian Wang (wkjian@xmu.edu.cn)

Graphic abstract of collection site and diseased fish

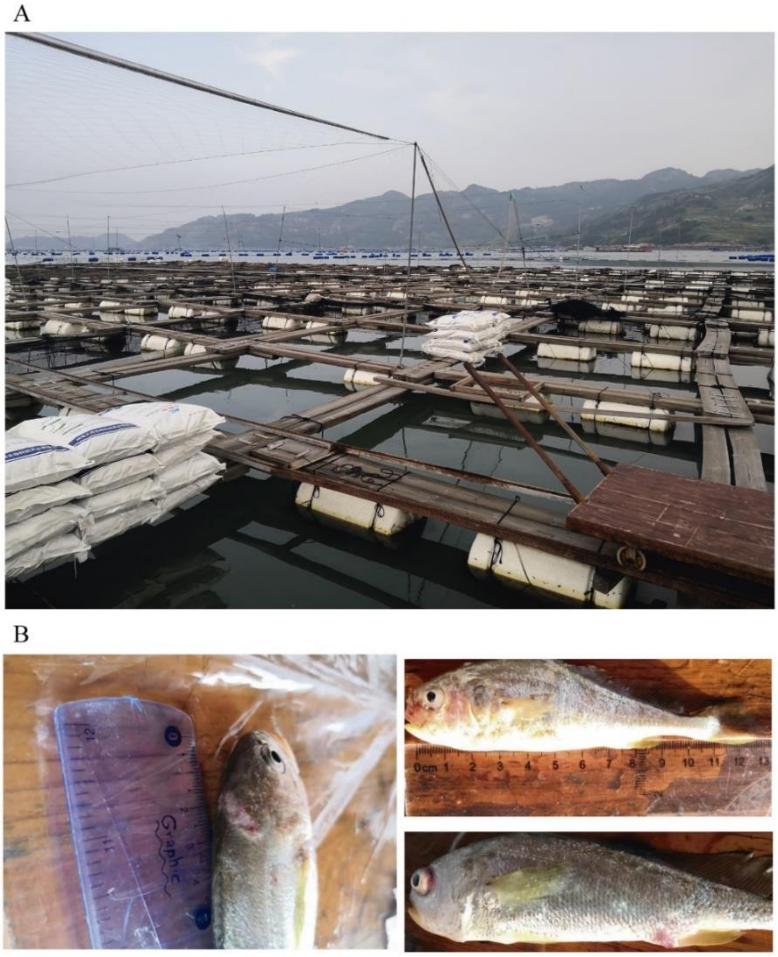

Figure S1. Graphic abstract of the collection site and diseased fish.
(A) The major production area for *L. crocea* aquaculture and spawning center in China is located at coastal regions under administration of Ningde city of Fujian Province. (B) We collected the dying fish which showed rotting lesions on the skin for further bacterial isolation.

Data of bacterial strains determined among isolates from large yellow croakers at 37 ℃

In the study, we also isolated bacterial strains at anaerobically and aerobically culture conditions at 27 ℃. Finally, we successfully isolated forty bacterial strains in different organs which could be isolated at 27 ℃. Summary data were shown in Table 1.

Table 1: Differential analysis of bacterial strains among all the isolates from diseased large yellow croakers at 37 ℃

Bacterial strains (Genus)	Organs				
	Intestine	Skin	Muscle	Gill	Tail fin
Hafnia	7				
Bacillus	3	3	4	1	2
Enterobacter				2	
Oceanbacillus	1				
Kocuria				1	
Staphylococcus		5	1	2	
Photobacterium	1			2	
Citrobacter	1			1	
Vibrio				1	
Enterococcus				1	
Ruegeria				1	

Data of bacterial strains determined among isolates from large yellow croakers at 27 ℃

In the study, we also isolated bacterial strains at anaerobically and aerobically culture conditions at 27 ℃. Finally, we successfully isolated twenty-four bacterial strains in different organs which could be isolated at 37 ℃. Summary data were shown in Table 2.

Table 2: Differential analysis of bacterial strains among all the isolates from diseased large yellow croakers at 27 ℃

Bacterial strains (Genus)	Organs			
	Intestine	Skin	Muscle	Gill
Bacillus	1	4	1	
Hafnia	8			
Staphylococcus	2	1	2	
Photobacterium	2			
Vibrio				2
Citrobacter				1

Evaluation of the purity and concentration for genomic DNA and DNA library

Agarose gel electrophoresis was conducted to assess the integrity and purity of the genomic DNA (Figure S2). Nanodrop and qubit were employed to measure the concentration of genomic DNA (Table 3) and the library reads (Table 4). Furthermore, pulsed field gel electrophoresis was carried out to assess the DNA library quality (Figure S3).

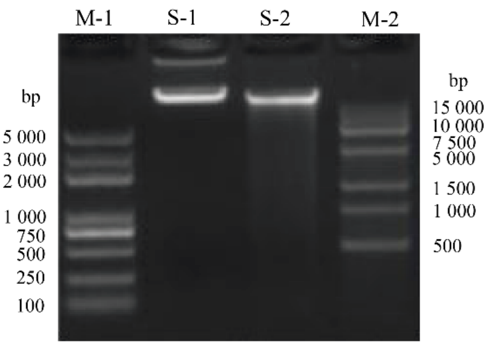

Figure S2. Agarose gel electrophoresis of genomic DNA. M-1 and M-2 panels represented two DNA markers Trans 2k plus and Trans 15k plus respectively. S-1 and S-2 panels represented the genome DNA samples with 5 µL and 0.5 µL accordingly.

Table 3. Evaluation of genomic DNA purity and concentration for *K. kristinae_LC*

Sample	Nanodrop Concentration (ng/μL)	260/280	260/230	Qubit Concentration (ng/μL)
K. kristinae_LC	215.1	1.88	1.9	108

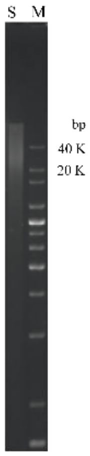

Figure S3. Pulsed field gel electrophoresis of library reads. M panel represented 1Kb DNA extension ladder and S panel was the reads sample with 0.5 μL.

Table 4. Evaluation of the quality of genomic DNA library construction for *K. kristinae_LC*

Standard for library construction for 20kb insert SMRTbell sequencing. Concentration≥100ng/uL, Total mass≥10ug, Main band≥30kb						
**Sample**	Concentration (ng/μL)	Total volume (μL)	Total mass (μg)	NC/QC	Distribution of DNA fragments (kb)	Type
K. kristinae_LC	108	263	28.40	1.99	≥30kb	10kb

Differential analysis of bacterial strains among all the isolates from diseased large yellow croakers

By analyzing and collective data of all the isolates from the organs, we found that the

top three genera bacteria are *Bacillus* spp., *Hafnia* spp., and *Staphylococcus* spp. with the percentages of 29.69, 23.44 and 20.31% accordingly (Table 5). And most the bacteria have reported to be zoonotic pathogens and cause infection in humans except the *Oceanbacillus* sp. and *Ruegeria* sp.

Table 5. Isolation percentages of isolates in each organ of the diseased large yellow croakers

Strain (Genus)	Number	Percentage
Hafnia	15	23.44%
Bacillus	19	29.69%
Enterobacter	2	3.13%
Oceanbacillus	1	1.56%
Kocuria	1	1.56%
Staphylococcus	13	20.31%
Photobacterium	5	7.81%
Citrobacter	3	4.69%
Vibrio	3	4.69%
Enterococcus	1	1.56%
Ruegeria	1	1.56%

Recovery of *K. kristinae_LC* from infected fish

We collected the livers and spleens of two infected fish at 48 h post challenge in an attempt to recover the investigated isolates. We washed and homogenized the tissues and then spread the solution on BHI plates for 16 hours at 37 ℃. The next day, we obtained a culture of the dominant bacteria, then selected colonies for further purification, and finally we carried out 16S rRNA gene sequencing. The strain was identified as *K. kristinae*. The result of the bacterial recovery was as shown in Figure S4.

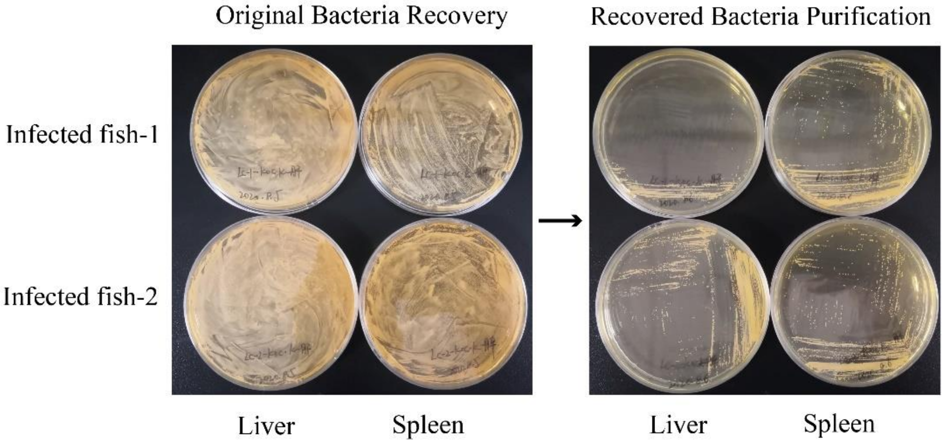

Figure S4. Process for *K. kristinae_LC* recovery from infected large yellow croakers.
